# Parity at last: a new funding model for undergraduate primary care education in England

**DOI:** 10.3399/bjgp22X719525

**Published:** 2022-05-27

**Authors:** Joe Rosenthal, Richard Darnton, Alex Harding

**Affiliations:** Department of Primary Care & Population Health, University College London, London.; Department of Public Health and Primary Care, University of Cambridge, Cambridge.; College of Medicine and Health, University of Exeter Medical School, Exeter.

Recruitment of medical graduates to general practice is a matter of national concern.^1^ Medical schools are recognised as critical to this mission,^2^ particularly given growing evidence to suggest that medical students’ experience of primary care is associated with their likelihood of choosing a GP career.^3^^–^^5^ Provision by medical schools of high-quality undergraduate GP teaching is also vital to the training of future secondary care specialists, who will no doubt in future be working increasingly in community-based and integrated care services. However, until now these important priorities in undergraduate medical education have been hampered by:

- chronic underfunding of undergraduate primary care clinical education relative to secondary care;
- systemic misunderstanding as to the nature and organisation of undergraduate GP teaching; and
- a lack of agency for GP educators with responsibility for leading and delivering undergraduate primary care teaching in medical schools.

On 31 March 2022, the Department of Health and Social Care in England (DHSC) published new education and training tariff guidance^6^ that finally goes some significant way to address these problems. This is an important and welcome step forward for the following reasons.

## 1) HISTORICAL FUNDING INEQUITIES BETWEEN PRIMARY AND SECONDARY CARE

This new undergraduate tariff introduces, for the first time, consistent national resourcing of medical student clinical teaching regardless of setting. To appreciate the significance of this development we should recall that until now the funding of medical student placements in general practice in England has not been included in the national education and training tariff but managed locally, based on an historical NHS payment system originally known as SIFT (service increment for teaching). This system has been widely regarded as outdated, inequitable, and in need of urgent review.^2^^,^^7^

When SIFT was first introduced in 1976 to *‘cover the additional service costs incurred by the NHS in providing facilities for the clinical teaching of medical students’*, it was paid only to teaching hospitals and was not available to the small number of GPs who then taught medical students.^8^ The Winyard report published in 1995 made SIFT available to general practices for the first time, initially at 12.5 GBP per half day session.^9^ No further national guidance on funding of GP teaching has been issued since Winyard. The 2012 consultation paper, *Liberating the NHS: Developing the Healthcare Workforce*, set out the Government’s commitment to a new system based on ‘tariffs’ for education and training as the foundation of a transparent funding regime that provides genuine incentives within the health sector.^10^

In 2013, following a formal impact assessment^11^ and a detailed cost collection in secondary care, the Department of Health in England did introduce a new tariff- based system for education and training in secondary care. SIFT was replaced by a national tariff paid to teaching hospitals in proportion to the number of students taught each year. The undergraduate tariff was initially set at 34 600 GBP per full-time equivalent (FTE) student per year, adjusted for each hospital by the NHS Market Forces Factor (MFF), an estimate of unavoidable cost differences between healthcare providers based on their location.

The 2013 costing exercise did not however include teaching in general practice, and the tariff system subsequently introduced did not apply to primary care. Student placements in general practice in England therefore continued to be funded based on historical SIFT and variable local arrangements, at a rate on average of two-thirds of the secondary care tariff.

The recent announcement of a harmonised undergraduate medical education and training tariff of 30 750 GBP plus MFF in all settings finally achieves parity for undergraduate education funding in general practice, albeit subsidised by a slight reduction in the payments allocated to secondary care placements.^6^

This harmonisation follows a detailed cost collection study published in the *BJGP* in 2019,^12^ which presented powerful evidence that the costs of providing undergraduate placements in general practice were considerably more than currently available funding, and broadly comparable to the higher funding allocated to placements in secondary care. Since publication of that study, extensive negotiations have taken place within the context of a National Tariff Advisory Group involving the DHSC, Health Education England (HEE), Society for Academic Primary Care, Royal College of General Practitioners, Medical Schools Council, British Medical Association, and Committee of General Practice Education Directors. The recommendations of this group went on to inform particularly Annex C of the new education and training tariff guidance document for the academic year 2022–2023.

## 2) PROVIDING A BESPOKE FUNDING MODEL FOR UNDERGRADUATE GP TEACHING

History is littered with organisational models created for secondary care being cookie-cuttered across to general practice with unsatisfactory results. During these latest tariff negotiations this risk was explicitly identified and hopefully averted. The resulting guidance recognises the unique nature of undergraduate general practice education. It supports the existing processes and controls developed over decades that already provide efficient, accountable use of clinical teaching funds and preclude inappropriate diversion from education into service delivery. The guidance acknowledges the requirement for a central GP team of GPs and administrators based within medical schools, who are responsible and accountable for all aspects of general practice education in the undergraduate medical course. It recognises the scale and complexity of their organisational responsibilities as well as the need for certain aspects of GP placements to be delivered centrally.

## 3) FORMALISING THE ROLE OF UNIVERSITY HEADS OF UNDERGRADUATE GP TEACHING

Responsibility without influence is never a comfortable space but this has until now been the situation for many of those who lead undergraduate GP teaching in England. The new education and training tariff guidance acknowledges their critical leadership role and provides them with a level of agency commensurate with their responsibilities. It does this by establishing them as the custodian of these funds, equipping them with the tools to oversee spending decisions, and giving them a seat alongside their medical course director colleagues at HEE regional inter-school liaison committees. In doing so, it formalises the role of the medical school head of undergraduate general practice teaching across the nation, similar to the way that the role of GP dean/director is now established in postgraduate education.

## THE FUTURE — RELATIVE UNDERFUNDING REMAINS A RISK

So, after a long journey, undergraduate GP teaching finally has a tailor-made funding model that provides parity with secondary care, and which formally supports the essential role of the head of undergraduate GP teaching and their team at each medical school.

Challenges, however, still lie ahead. Parity of primary and secondary care education funding is necessary but may not prove to be sufficient. For example, transporting students from universities to more remote GP practices, and accommodating them while they are there, is expensive and now exposed to worrying price inflation. Geographical dispersion is central to providing students with an authentic experience of general practice, and encouraging them to consider living and working in rural and remote areas themselves one day.

Undergraduate medical tariff has been set for all at 30 750 GBP per FTE student per year for 2022–2023,^6^ but if this figure does not increase annually with inflation, undergraduate GP teaching, given its high exposure to market cost pressures, will be the first to suffer. While we can now celebrate a new funding model for medical student GP teaching, suitable funding levels must also be maintained in the future if it is to achieve its long-term purpose.
